# Heavy Cigarette Smokers in a Chinese Population Display a Compromised Permeability Barrier

**DOI:** 10.1155/2016/9704598

**Published:** 2016-06-29

**Authors:** Shujun Xin, Li Ye, George Man, Chengzhi Lv, Peter M. Elias, Mao-Qiang Man

**Affiliations:** ^1^Dalian Skin Disease Hospital, Dalian, Liaoning 116021, China; ^2^Dermatology Services, San Francisco Veterans Affairs Medical Center and University of California San Francisco, San Francisco, CA 94121, USA

## Abstract

Cigarette smoking is associated with various cutaneous disorders with defective permeability. Yet, whether cigarette smoking influences epidermal permeability barrier function is largely unknown. Here, we measured skin biophysical properties, including permeability barrier homeostasis, stratum corneum (SC) integrity, SC hydration, skin surface pH, and skin melanin/erythema index, in cigarette smokers. A total of 99 male volunteers were enrolled in this study. Smokers were categorized as light-to-moderate (<20 cigarettes/day) or heavy smokers (≥20 cigarettes/day). An MPA5 was used to measure SC hydration and skin melanin/erythema index on the dorsal hand, forehead, and cheek. Basal transepidermal water loss (TEWL) and barrier recovery rates were assessed on the forearm. A Skin-pH-Meter pH900 was used to measure skin surface pH. Our results showed that heavy cigarette smokers exhibited delayed barrier recovery after acute abrogation (1.02% ± 13.06 versus 16.48% ± 6.07), and barrier recovery rates correlated negatively with the number of daily cigarettes consumption (*p* = 0.0087). Changes in biophysical parameters in cigarette smokers varied with body sites. In conclusion, heavy cigarette smokers display compromised permeability barrier homeostasis, which could contribute, in part, to the increased prevalence of certain cutaneous disorders characterized by defective permeability. Thus, improving epidermal permeability barrier should be considered for heavy cigarette smokers.

## 1. Introduction

Cigarette smoke negatively impacts human health in multiple organ systems. Previous studies have shown that cigarette smoking inhibits not only sympathetic nerve activity [1, 2] but also innate immune responses [3–5]. Clinical studies have also demonstrated that cigarette smoke is a risk factor for developing cardiovascular diseases [6, 7] and for death from subclinical coronary atherosclerosis [8]. Additionally, cigarette smoke also increases the risk for developing cancers [9–11] and hip fractures [12–14]. Likewise, cigarette smoking damages renal and liver functions [15, 16]. Moreover, in utero exposure to cigarette smoking reduces not only the birth weight and length of newborn babies [15], but also lung function, including tidal flow-volume ratios [17–19]. In a murine model, newborn mice also display both liver and spleen abnormalities when their mothers are exposed to cigarette smoke during pregnancy [20]. Finally, altered innate immune responses to both bacterial and viral infections have also been demonstrated following prolonged exposure to cigarette smoke [21–23].

Cigarette smoke also impacts cutaneous function. Cigarette smoke delays cutaneous wound healing [24, 25] and elevates dermal matrix metalloproteinase-8 levels [24]. Epidemiological studies have shown that cigarette smoking increases the incidence of psoriasis [26, 27] and the risk of developing psoriasis is proportional to both the dosage and duration of smoking [27]. Likewise, second-hand cigarette smoking is a significant risk factor for the development of atopic dermatitis in children [28–31].

The development of psoriasis and atopic dermatitis is associated with defective epidermal permeability barrier function [32, 33]. Studies have demonstrated that nicotine, a major constituent in cigarettes, stimulates keratinocyte differentiation via inducing calcium influx in vitro [34, 35], but calcium influx also delays epidermal permeability barrier recovery [36]. While these studies together suggest that cigarette smoke compromises epidermal function, the relationship has not yet been assessed. In the present study, we assessed a suite of epidermal functions in smokers versus nonsmokers in a Chinese population.

## 2. Subjects and Methods

### 2.1. Subjects

A total of 99 male volunteers, including 63 smokers and 36 nonsmokers, aged 41–65 years (mean 53.86 ± 0.66) were enrolled in this study. All were indoor workers with no current skin or systemic diseases, which could influence epidermal barrier function. Smokers were categorized as light-to-moderate (<20 cigarettes/day) or heavy smokers (≥20 cigarettes/day). The clinical characteristics of the subjects are detailed in Table 1. All smokers had smoked for at least 2 years, and were still smoking at the time of the study. No skin care products were applied to measured sites 24 hour prior to taking measurements and the measured sites were not washed with soaps or surfactants for at least 12 hours prior to study.

### 2.2. Measurements

All measurements were randomly performed by two fully trained dermatologists. TEWL and SC electrical capacitance were measured on the right dorsal hand, right cheek, and forehead with respective probes (TM300 and Corneometer CM825) attached to a Courage-Khazaka MPA5 system [37–39]. For stratum corneum integrity assessment, TEWL was measured on the forearm following each D-Squame application for a total of 6 D-Squames [37]. For barrier recovery, barrier disruption was achieved by repeated D-Squame applications for a total of 6 D-Squames. TEWL was measured both immediately and 3 hours after the last D-Squame application. A Skin-pH-Meter pH900 was used to measure skin surface pH, and melanin/erythema probe connected to MPA5 was used to measure melanin/erythema index. All subjects rested for at least 30 min at 22–24°C at a relative humidity of 45–55%, prior to measurement. This work was performed between October and March at Dalian Skin Disease Hospital, China. The study adhered to the ethical guidelines of the Declaration of Helsinki.

All subjects have been given their informed consent.

### 2.3. Statistics

GraphPad Prism 4 software was used for all statistical analyses. An unpaired *t*-test with Welch's correction was used for comparisons between two groups. A one-way ANOVA Kruskal-Wallis test with Dunn's multiple comparison was used to determine significances when three or more groups were compared. Linear regression was used to determine the correlation between barrier recovery rates and the amount of daily cigarette consumption. Data are expressed as mean ± SEM.

## 3. Results 

### 3.1. Heavy Cigarette Smokers Display a Compromised Epidermal Permeability Barrier Homeostasis

**Figure 1:**
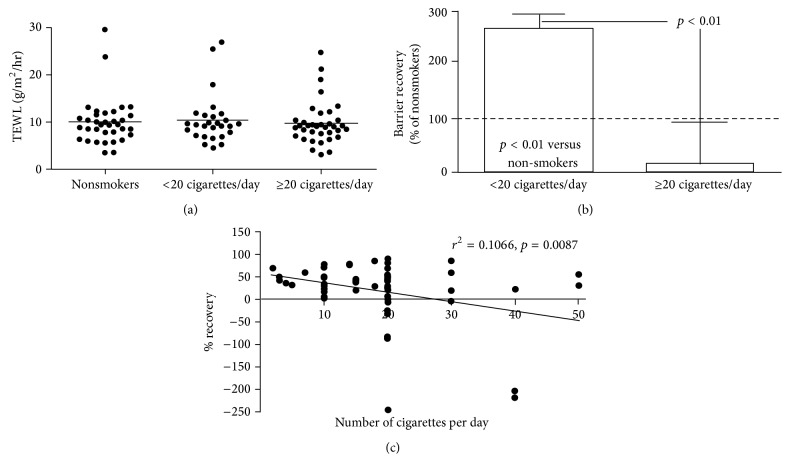
Changes in epidermal permeability barrier function in cigarette smokers. Basal TEWL and barrier repair kinetics on the forearm were determined as described in Subjects and Methods. A one-way ANOVA Kruskal-Wallis test was used to determine significant differences as shown in (a) and (b). (c) shows correlation between barrier recovery rates and the number of cigarettes smoked. The number of subjects in each group is described in Table 1.

**Figure 2:**
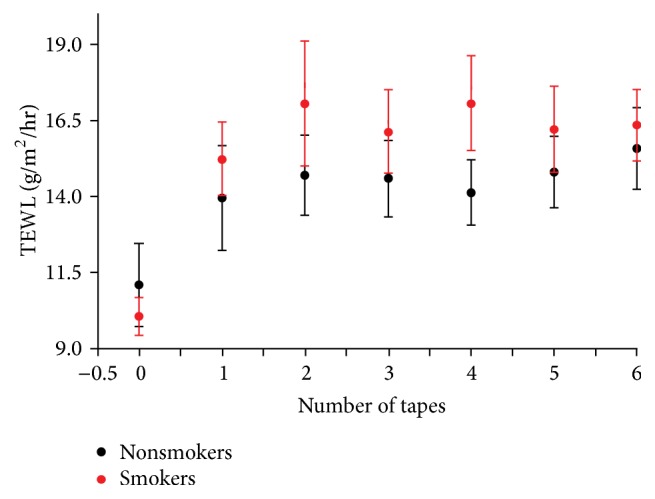
Comparison of stratum corneum integrity between smokers and nonsmokers. Stratum corneum integrity on the forearm was assessed by measuring TEWL after each D-Squame application. A total of 6 D-Squames were applied. An unpaired two-tailed Student's *t*-test was used to determine significant differences between smokers and nonsmokers. The number of subjects in each group is detailed in Table 1.

We first assessed epidermal permeability barrier homeostasis on the forearm of smokers versus nonsmokers. While there were no differences in basal TEWL between smokers and nonsmokers (Figure 1(a)), permeability barrier recovery accelerated in light cigarette smokers in comparison to nonsmokers (Figure 1(b). *p* < 0.01), but barrier repair kinetics instead were delayed in heavy cigarette smokers (Figure 1(b)). Notably, permeability barrier recovery rates correlated negatively with the extent of cigarette consumption (Figure 1(c)), but neither basal TEWL nor permeability barrier recovery rates correlated with the number of years that subjects had been smoking (data not shown). Stratum corneum integrity on the dorsal hand was similar between smokers and nonsmokers (Figure 2). Similarly, neither the number of years that subjects had been smoking nor the number of cigarettes smoked daily correlated with stratum corneum integrity (data not shown). These results indicate that heavy cigarette smoking compromises epidermal permeability barrier homeostasis.

### 3.2. Alterations in Skin Surface pH and Stratum Corneum Hydration on Cigarette Smokers

**Figure 3:**
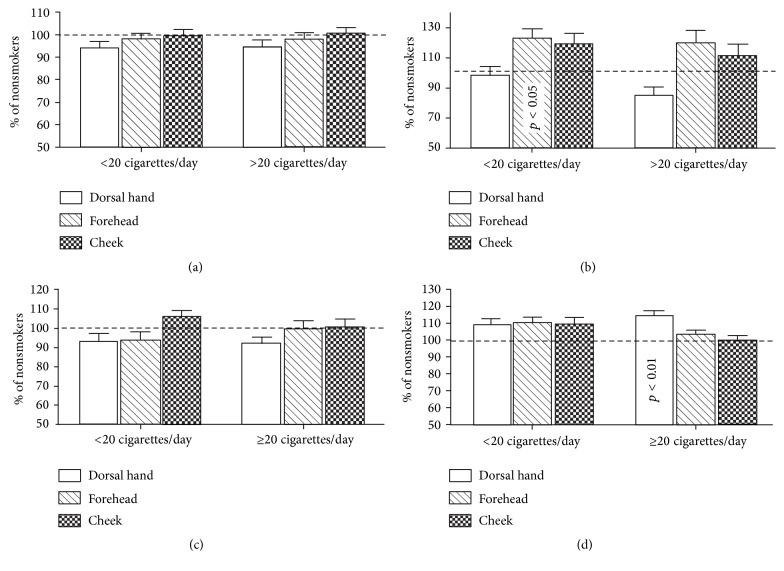
Comparison of epidermal biophysical properties between smokers and nonsmokers. (a) and (b) depict skin surface pH and stratum corneum hydration, respectively, while (c) and (d) display the melanin and erythema index, respectively. The number of subjects in each group is detailed in Table 1. One-way ANOVA Kruskal-Wallis test was used to determine significant differences between smokers and nonsmokers, and the significances are indicated in the figures.

Since the permeability barrier is closely associated with changes in skin surface pH and stratum corneum hydration [40–42], we next compared skin surface pH and stratum corneum hydration in smokers versus nonsmokers. While there were no differences in skin surface pH between smokers and nonsmokers (Figure 3(a)), stratum corneum hydration on the forehead was significantly higher in light smokers than in nonsmokers (Figure 3(b)). Aside from a significantly higher erythema index on the dorsal hand of heavy cigarette smokers (Figure 3(d)), there were no significant differences in either melanin or erythema indices between smokers and nonsmokers. These results demonstrate that changes in certain skin biophysical properties vary with both body site and the number of cigarettes smoked daily.

## 4. Discussion

Cigarette smoke is a major public health issue, because it is associated with the pathogenesis of a variety of clinical disorders [6–12]. Although the impact of cigarette smoking on certain systemic functions has been extensively studied, the impact of smoking on epidermal permeability barrier function has not yet been well characterized. In the present study, we demonstrated that while basal permeability barrier function remains unchanged, heavy cigarette smokers display altered epidermal permeability barrier homeostasis. Moreover, permeability barrier repair kinetics correlated negatively with the number of cigarettes smoked daily. However, in contrast to the our findings, Muizzuddin et al. found higher basal TEWL levels in cigarette smokers than in nonsmokers [43]. This discrepancy with our data could be attributed to body site differences. It has been demonstrated that skin physiology, including permeability barrier function, vary with the body sites in response to external stressors [44, 45]. While Muizzuddin et al. measured TEWL on the cheek, we measured TEWL on the forearm. Nevertheless, both studies clearly show that cigarette smoking is associated with compromised permeability barrier homeostasis.

The mechanisms by which cigarette smokers display such diverse differences in permeability barrier homeostasis are not clear. But several studies have shown that nicotine, a major ingredient in cigarettes, regulates keratinocyte functions. Addition of nicotine to keratinocyte cultures increases filaggrin and involucrin expression [46, 47] and enhances the formation of cornified envelopes [48]. Moreover, nicotine stimulates keratinocyte adhesion [49]. Administration of nicotine increases cholesterol production in rats [50–52]. These effects of nicotine benefit permeability barrier [35, 53–56]. Thus, these benefits induced by nicotine could be attributable to improved barrier homeostasis in light cigarette smokers. On the other hand, nicotine also negatively impacts keratinocyte functions, including the stimulation of both cytokine release [57–59] and oxidative stress [60]. While cytokines can benefit barrier repair [61, 62], if excessive inflammation occurs, it can impair epidermal permeability barrier function [63, 64]. Likewise, oxidative stress inhibits keratinocyte proliferation and differentiation, as well as HDL receptor expression [65–67], which could result in a defective epidermal permeability barrier homeostasis. Thus, it is likely that the beneficial effects of nicotine-induced keratinocyte differentiation are overridden by the negative effects of excessive amount of cigarette smoking. Clinically, the negative impact of cigarette smoking on epidermal permeability barrier function has significant consequences. A compromised permeability barrier predisposes one to develop dermatitis and psoriasis [32, 33, 54, 68], both of which are associated with certain systemic disorders such as diabetes and cardiovascular diseases [69–72].

The present study also demonstrates that stratum corneum hydration is high on sebaceous gland-enriched sites (the forehead and cheeks) of smokers. This change could reflect increased sebum production, induced by nicotine. A previous study showed that nicotine stimulates sebocyte proliferation and lipid production [73], and we have shown that sebum-originated glycerol is a key determinant of stratum corneum hydration [74, 75]. In contrast to sebaceous gland-enriched sites, stratum corneum hydration is lower on the dorsal hand of heavy smokers. This could be due to the toxic effects induced by nicotine, because high concentrations of nicotine can inhibit keratinocyte proliferation and protein synthesis [47, 48]. Collectively, nicotine stimulates sebum production while inhibiting keratinocyte proliferation and protein synthesis. Thus, cigarette smokers display a higher stratum corneum hydration on sebum-enriched sites and a lower stratum corneum hydration on sebum-impoverished sites.

In summary, the present study shows that changes in epidermal permeability barrier homeostasis correlate with the number of cigarettes smoked daily, suggesting a pathogenic role of cigarette smoking in the development of certain cigarette-associated disorders. Therefore, the improvement of epidermal permeability barrier is important for cigarette smokers, particularly heavy smokers.

## Figures and Tables

**Table 1:** Clinical characteristics of subjects.

Group	*N*	Age	Years of smoking	Number of cigarettes/day
Nonsmokers	36	53.00 ± 1.20	N/A	N/A
All smokers	63	54.21 ± 0.81	28.79 ± 1.16	18.03 ± 1.15
≥20 cigarettes/day	36	54.72 ± 1.04	30.28 ± 1.18	23.61 ± 1.27
<20 cigarettes/day	27	53.52 ± 1.26	26.81 ± 2.17	10.59 ± 0.88
